# Development of nanocubosomes co-loaded with dual anticancer agents curcumin and temozolomide for effective Colon cancer therapy

**DOI:** 10.1080/10717544.2022.2108938

**Published:** 2022-08-08

**Authors:** Yosif Almoshari, Haroon Iqbal, Anam Razzaq, Khalil Ali Ahmad, Muhammad Khalid Khan, Saad Saeed Alqahtani, Muhammad Hadi Sultan, Barkat Ali Khan

**Affiliations:** a Department of Pharmaceutics, College of Pharmacy, Jazan University, Jazan, Saudi Arabia; b The Cancer Hospital of the University of Chinese Academy of Sciences (Zhejiang Cancer Hospital) Institute of Basic Medicine and Cancer (IBMC), Chinese Academy of Sciences Hangzhou, Zhejiang, China; c College of Pharmaceutical Sciences, Soochow University, Suzhou, China; d Drug Delivery and Cosmetic Lab (DDCL), Gomal Center of Pharmaceutical Sciences, Faculty of Pharmacy, Gomal University, Dera Ismail Khan, Pakistan; e Department of Clinical Pharmacy, College of Pharmacy, Jazan University, Jazan, Saudi Arabia

**Keywords:** Nanocubosomes, curcumin, temozolomide, colon cancer, therapy

## Abstract

Current research aimed to develop nanocubosomes co-loaded with dual anticancer drugs curcumin and temozolomide for effective colon cancer therapy. Drugs co-loaded nanocubosomal dispersion was prepared by modified emulsification method using glyceryl monooleate (GMO), pluronic F127 and bovine serum albumin (BSA) as a lipid phase, surfactant, and stabilizer, respectively. The resulting nanocubosomes were characterized by measuring hydrodynamic particle size, particle size distribution (PSD), drug loading capacity (DL), encapsulation efficiency (EE), colloidal stability and drug release profile. We also physiochemically characterized the nanocubosomes by transmission electron microscopy (TEM), Fourier transform infrared (FTIR), and x-rays diffraction (XRD) for their morphology, polymer drug interaction and its nature, respectively. Further, the *in-vitro* cell-uptake, mechanism of cell-uptake, *in-vitro* anti-tumor efficacy and apoptosis level were evaluated using HCT-116 colon cancer cells. The prepared nanocubosomes exhibited a small hydrodynamic particle size (PS of 150 ± 10 nm in diameter) with nearly cubic shape and appropriate polydispersity index (PDI), enhanced drug loading capacity (LC of 6.82 ± 2.03% (Cur) and 9.65 ± 1.53% (TMZ), high entrapment efficiency (EE of 67.43 ± 2.16% (Cur) and 75.55 ± 3.25% (TMZ), pH-triggered drug release profile and higher colloidal stability in various physiological medium. Moreover, the nanocubosomes showed higher cellular uptake, *in-vitro* cytotoxicity and apoptosis compared to free drugs, curcumin and temozolomide, most likely because its small particle size. In addition, BSA-stabilized nanocubosomes were actively taken by aggressive colon cancer cells that over-expressed the albumin receptors and utilized BSA as nutrient source for their growth. In short, this study provides a new and simple strategy to improve the efficacy and simultaneously overawed the adaptive treatment tolerance in colon cancer.

## Introduction

Adaptive treatment tolerance (ATT) is considered as one of the major obstacles in cancer therapy, which causes poor sensitivity in cancer cells to the chemotherapeutic drugs due to prolonged exposure (Zhao et al., 2014; Motevalli et al., 2019). ATT might be triggered by three processes, (i) the drugs failed to cross the plasma membrane (ii) the drugs easily exorcize from lysosomal environment of the cancer cells (iii) the activation of efflux pump or multidrug resistance (MDR) proteins which transport or escape the drugs from the cancer cells (Housman et al., 2014; Zhitomirsky & Assaraf, 2016). One potential strategy applied to overawed ATT is the administration of two or more chemotherapeutic drugs to the cancer cells together (C. Wang et al., 2013;). Chemotherapeutic drugs can be co-administered into cancer cells by two different approaches, i.e. sequential and simultaneous co-administration (Poradowski & Chrószcz, 2022). In sequential co-administration approach, cancer cells were first treated with chemosensitizers followed by the chemotherapeutic drug administration. Pretreatment of chemosensitizers significantly upsurges the efficiency of chemotherapeutic drugs in cancer cells (Benyettou et al., 2017). For example, erlotinib pretreatment blocked the epidermal growth factor (EGF) receptor and enhanced the therapeutic activity of doxorubicin against breast cancers (Lee et al., 2012). However, sequential approach is not always preferred for the co-administration of anticancer drugs. In contrast, simultaneous co-administration approach of chemotherapeutic drugs shows more therapeutic efficacy in advanced and resectable colorectal tumors as compared to sequential approach of co-administration for chemotherapeutic drugs (Ducreux et al., 2011; Fares et al., 2020). However, co-administration of chemotherapeutic drugs usually fails to attain its full therapeutic efficacy due to its poor water solubility, rapid clearance, and off-target toxicities. To combat this problem, chemotherapeutic drugs have been co-loaded into nanodrug delivery systems, which can enhance the therapeutic activity of chemotherapeutic drugs by enabling their enhanced cell penetration and intracellular distribution, and reducing their toxic effects on healthy cells (Bronze-Uhle et al., 2017; Li et al., 2021; Sohail et al., 2022).

Recently, nanocubosomes have been emerged as a novel nanodrug delivery system and considered as a substitute nanocarriers system to liposomes due to their inherent potential to encapsulate both hydrophilic and lipophilic drugs (Karthikeyan, 2017; Patra et al., 2018). Nanocubosomes, comprised of binate systems of glyceryl monooleate (GMO) and water the most widely investigated systems. Nanocubosomes are characterized by their large interior surface area and viscid isotropic nature, having the ability to self-assembled into cubic liquid crystalline nanosystem (Al-Mahallawi et al., 2021). These liquid crystalline nanosystems have been applied as drug carrier due to their unique three-dimensional (3-D) structure with lipophilic and hydrophilic subunits (Tu et al., 2014). The encapsulated drug molecules could be released in controlled pattern due to the large interior surface area which provide complex diffusion pathways. Furthermore, the lipid subunits GMO are biodegradable, biocompatible, and bioadhesive (Patra et al., 2018; Al-Mahallawi et al., 2021). Thus, nanocubosomes have been exploited as versatile nanocarriers for the delivery of chemotherapeutics, photosensitizers, peptides, enzymes, and antibiotics.

Temozolomide (TMZ) is a broad-spectrum DNA alkylating chemotherapeutic drug effectively used in the treatment of brain tumor, metastatic melanoma and colon cancer (Waghule et al., 2021). TMZ has short half-life (1.8 h) and undergoes a spontaneous hydrolysis to (3-methyltriazen-1-yl) imidazole-4-carboxamide (MTIC) at normal physiological pH (Yasaswi et al., 2021). MTIC metabolite rapidly converts (half-life 2.5 min) into methyl diazonium ion and 5-amino-imidazole-4-carboxamide responsible for the alkylation of guanine residue in DNA strand and subsequently induce DNA damage (R. Wang et al., 2022). Howbeit, these active alkylating species have poor penetration and deep tumor permeation. Thus, higher concentration and repeated doses of TMZ are needed, leading to thrombocytopenia, myelosuppression, neutropenia and drug resistance (Zhang et al., 2022). So, these limitations can be tackled by encapsulating TMZ into nanodrug delivery system to enhance the biological half-life and avoid the exposure to systemic pH and also the co-treatment of chemosensitizers. In current study, we explored the co-treatment of HCT-116 colon cancer cells with TMZ and curcumin (Cur) a chemosensitizer. Cur is natural small molecule with diverse properties such as antioxidant, anti-amyloid, anti-arthritic, anti-inflammatory, anti-ischemic and antitumor (Mehanny et al., 2016a; Tomeh et al., 2019; Baldi et al., 2020; Rubab et al., 2021). In addition, Cur also inhibits the P-glycoprotein (P-gp) an efflux protein expressed colon epithelial cells, and inhibits the transport of chemotherapeutic drugs from interior of cancer cells (Sagnou et al., 2020). Inhibition of colon Pgp shows a substantial clinical effect on the intra-cellular concentration of chemotherapeutic drug. Herein, we co-loaded dual anticancer drugs TMZ and Cur into nanocubosomes (self-assembled crystalline cubic liquid nanoparticles) to upsurge efficacy against HCT-116 colon cancer cells, reduced the side effects and improve the patient compliance.

## 2. Materials and methods

### 2.1. Materials

Temozolomide (TMZ) (99.9% purity), glyceryl monooleate (GMO) and freez (Poloxamer 407) were purchased from ACEM biochemical, Shanghai, China. Bovine serum albumin and curcumin were obtained from BioFroxx Germany. The primary antibodies for P-gp (22336-1-AP) and GAPDH (60004-1-Ig) were purchased from proteintech (MA, USA), while secondary antibodies anti-rabbit IgG (7074S) and anti-mouse IgG (7076S) were obtained from Cell Signaling Technology, Inc.

### 2.2. Preparation of nanocubosomes

Self-assembled blank and drug loaded nanocubosomes were prepared by modified emulsification method (Morsi et al., 2014). Briefly, GMO (50 mg) and Pluronic-F127 (25 mg) were heated at 70 ± 5 °C for 30 min. Next, the molten solution was drop-wise added to 10 mL distilled water containing BSA (10 mg) at 55 ± 2 °C under continuous stirring at 1000 rpm. The obtained dispersions were stirred for further 2 h and then cooled down to room temperature (25 ± 2 °C) to achieve a homogenous mixture followed by ultra-probe sonication for 5 min for two cycle (2.5 min/cycle) at 65% amplitude and allow the emulsion to grow at room temperature to solidify the lipid droplets. For drug loaded nanocubosomes, Cur (5 mg) and TMZ (10 mg) were supplied to the oily phase and aqueous phase, respectively prior to mixing. We use univariate one variable at a time (OVAT) method to get the optimized formulation (Data not shown). The prepared CTNCs were suspended in a 2% sucrose solution (w/v), lyophilized (LyoQuest, Telstar, Shangai, China) and subsequently stored the lyophilized NPs at 4 °C prior to further use.

### 2.3. Characterization of nanocubosomes

#### 2.3.1. Measurement of hydrodynamic diameter and surface charge

The average hydrodynamic diameter/particle size of nanocubosomes, their distribution (polydispersity index, PDI) in aqueous medium was measured by particle size analyzer (Malvern Zetasizer Nano Ver. 6.20, Malvern, UK) at scattering angle (90◦) with 60 sec equilibrium time between the cycles. The surface zeta potential value of nanocubosomes were evaluated by applying voltage across a pair of electrodes both end of cell containing the nanocubosomes dispersion. All samples were analyzed in triplicate independently.

#### 2.3.2. Surface morphology

The surface morphology of nanocubosomes were envisioned by TEM (Joel JEM 1230, Tokyo, Japan). One drop (5 µL) of diluted nanocubosomal dispersion was placed on copper grid followed by negative staining with uranyl acetate (2% v/v) for 2 min and allow to dry at room temperature before imaging under TEM.

#### 2.3.3. Drug loading and encapsulation efficiency

The percent drug loading (DL %) and entrapment efficiency (EE %) Cur/TMZ nanocubosomes were measured by extracting Cur and TMZ using methanol as solvent and analyzed by UV spectrophotometer (Shimadzu UV-2600 Spectrometer, Kyoto, Japan) at 429 nm and 329 nm, respectively and amount of each drug was calculated by using their respective standard curve. The DL % and EE % was calculated as follows:

$$\mathrm{DL}\;\%=\frac{\mathrm{Conc}.\text{ of Cur}/\text{TMZ in NPs}}{\text{Weight of dried NPs}}\times 100$$

$$\mathrm{EE}\;\%=\frac{\mathrm{Conc}.\text{ of Cur}/\text{TMZ in NPs}}{\mathrm{Cur}/\text{TMZ conc}.\text{ initially added}}\times 100$$

#### 2.3.4. Fourier transform infrared (FTIR) analysis

The presence of representative functional groups and intramolecular interactions amid the drugs and GMO in fabricated nanocubosomes were analyzed by FTIR spectroscopy (Bruker, Karlsruhe, Germany) (Iqbal et al., 2021; Khan et al., 2022). Briefly, a small quantity of Cur, TMZ, CTNCs and blank nanocubosomes freeze-dried powder were directly placed on sample chamber and IR spectrum was measured in the IR range (500–4000 cm^−1^), with 2 mm sec^−1^ scanning speed for 120 scans with 4 cm^−1^ resolution.

#### 2.3.5. X-ray diffraction (XRD) analysis

The X-ray spectrum of Cur, TMZ, CTNCs and blank nanocubosomes were recorded by D8 ADVANCE diffractometer (Bruker, Karlsruhe Germany), operating system of 40 mA current, 40 KV voltage and 1.5406 Å specific wavelength with 0.02° scan step size and 17.7 sec scan step time (Aditya et al., 2015).

#### 2.3.6. Differential scanning calorimetry (DSC) analysis

DSC spectrum of Cur, TMZ, Cur/TMZ nanocubosomes and blank nanocubosomes were recorded using simultaneous thermal analyzer (STA449F3, Netzsch, Bavaria, Germany). Each freeze-dried sample (1–2 mg) was heated in an aluminum sealed pan at a heating rate of 10°Cmin^−1^ from 80 to 220 °C with continuous nitrogen flow (30 mL min^−1^). A blank aluminum sealed pan was applied as reference control (Shah et al., 2019).

#### 2.3.7. Colloidal stability

The colloidal or dispersion stability of CTNCs were evaluated in different physiological medium (PBS, D/W, 0.9% NaCl, 5% glucose and RPMI-1640) using previously reported protocol (Rohiwal et al., 2015; Valdeperez et al., 2022). Briefly 0.1 mg freeze-dried CTNCs were dispersed in 1 mL of each stated medium and kept at 4 °C refrigerator. Next, the hydrodynamic diameter and surface zeta potential of each sample was measured in triplicate at specified time intervals (0, 3, 6, 12, 24, 48, 96 and 120 h) using particle size analyzer.

#### 2.3.8. *In-vitro* drug release

The *in-vitro* release of Cur and TMZ from the CTNCs were performed by dialysis bag method using release medium with pH 7.4 and 5.5 mimicking the normal and tumor pH respectively (Kudarha & Sawant, 2021). Briefly, CTNCs suspension (1 mL) with respect to Cur and TMZ concentration of 150 and 220 µg/mL, respectively were sealed in dialysis bag (Solarbio, Bejing, China) of 8000–14000 Da molecular cut off. The dialysis bags were suspended in 15 mL centrifuged tube supplemented with 10 mL release medium containing PEG 400 (30% V/V) and shake the centrifuge tube with speed of 150 at 37 °C. At specified time intervals (0.5, 1.0, 3.0, 6.0, 12.0, 24.0, and 48.0 h) withdrawn 2 mL of sample aliquot and replenished the same volume of fresh medium. The amount of Cur and TMZ released from CTNCs were quantified by *in vitro UV* spectroscopic method.

### 2.4. *In vitro* cell studies

Human colon cancer (HCT-116) cell line was supplied by cell bank (Shanghai, China). The cells were maintained under optimal growth condition (5% CO_2_, 37 °C) and further grow in complete RPMI-1640 supplied with fetal calf serum (FCS) 10% and 1% antibiotics (penicillin & streptomycin).

#### 2.4.1. Cell uptake

The cell uptake of CTNCs were evaluated in HCT-116 colon cancer cell using our previously reported protocol with minor modifications (H. Iqbal et al., 2021). Briefly, HCT-116 cells were seeded at a cell density of 1 × 10^5^ cells/well in 1.0 mL complete RPMI-1640 with 10% FCS. After 12 h growth, the cells were incubated with CTNCs, Cur and TMZ (2.0 µg/mL) for different time periods (4, 6, 12 and 24 h), to analyzed the time-dependent cell uptake of Cur and TMZ in CTNCs. Untreated cells in each plate was used as reference or control group. Afterward, the treated cells were carefully washed with cold PBS, detach the cells with Trypsin/EDTA (0.5 mM) from the plates, pelleted the cells by centrifugation at 3000 rpm, and dispersed in cold PBS (2.0 mL). Eventually, the cells were counted by automatic cell counter (Countess II FL, Invitrogen) and lysis the cells using ultrasonic prob sonicator (BAOSHISHAN FS-600N, USA) and finally extract the Cur and TMZ using methanol as a solvent. Afterwards, the concentration of Cur and TMZ was quantified by UV sphectrophometer (Floustar Omega, Germany) at 429 nm and 329 nm, respectively and the amount of each drug was calculated by using their respective standard curve. The experiment was triplicated, and the data are expressed as an average value with SD.

#### 2.4.2. Mechanism of cell uptake

To assess the endocytic pathway or cell uptake mechanism of CTNCs, HCT-116 cells (1.0 × 10^5^) were plated in 1.0 mL RPMI-1640 with 10% FCS for 24 h in each well. After 24 h growth, cells were aspirated, washed with cold PBS and supplied a fresh medium followed by inhibition with various inhibitors such as chlorpromazine (10.0 µg/mL, inhibiting clathrin-mediated cell uptake), amiloride (100.0 µg/mL, inhibiting macropinocytosis), nystatin (2.0 µg/mL, inhibiting caveolae-mediated cell uptake), and albumin (1 mg/mL, inhibiting albumin receptor mediated endocytosis) for 0.5 h. After inhibitors treatment, the cells in each well was carefully washed with PBS, and further treated with CTNCs (2.0 µg/mL with respect to TMZ) and incubated for 12 h. To evaluate the active and passive transport mechanism, HCT-116 cells were incubated at 37 °C 4 °C and for 12 h. Next, the treated cells were washed with cold PBS, detach the cells with Trypsin/EDTA (0.5 mM) from the plates, pelleted the cells by centrifugation at 3000 rpm, and dispersed in cold PBS (2.0 mL). Eventually, the cells were counted by automatic cell counter (Countess II FL, Invitrogen) and lysis the cells using ultrasonic prob sonicator (BAOSHISHAN FS-600N, USA) and finally extract the Cur and TMZ using methanol as a solvent. Afterwards, the concentration of Cur and TMZ was quantified by UV sphectrophometer (Floustar Omega, Germany) at 429 nm and 329 nm, respectively and the amount of each drug was calculated by using their respective standard curve. The experiment was triplicated, and the data are expressed as an average value with SD.

#### 2.4.3. *Intra-cellular* distribution

Intra-cellular distribution of CTNCs in HCT-116 cells were evaluated by confocal microscopy. Briefly, HCT-116 cells (5 × 10^4^) were cultured in a glass bottom confocal imaging dish and incubated in 5% CO_2_ incubator for 12 h. Next, the cells were incubated with CTNCs, Cur and TMZ (5.0 µg/mL with respect to Cur and TMZ), and further grow at 37 °C for 4 h. After treatment, the medium was aspirated, three-times washed the cells with PBS and stained with Hoechst (5.0 µg/mL) for 5 min, followed by careful washing with PBS five time. Afterward, the cells were stained with lysotracker red for 10 min. Finally, the stained cells were carefully washed five time with PBS and analyzed by confocal laser scanning microscope (CLSM 510 META).

#### 2.4.4. *In-vitro* anticancer efficacy

The *in-vitro* cytotoxicity of CTNCs were evaluated by SRB assay (Saber et al., 2018). Briefly, the exponentially growing HCT-116 cells (5 × 10^3^) were seeded in each well of 96-well plates and allow for overnight growth. Next the cells were treated with CTNCs, and mixture of free Cur and TMZ at different concentration range (1.5625, 3.125, 6.25, 12.5, 25, 50, 100 µg/mL) and incubated for further 24 h and 48 h to determine the IC_50_ value (drug concentration that inhibit 50% cell growth). After treatment, cells were riveted with 10% trichloroacetic acid (C_2_HCl_3_O_2_) at 4 °C for 1 h followed by staining with 0.4% SRB reagent for 30 min and subsequently washed the cells three times with acetic acid (1%) aqueous solution and air-dried. Afterwards, Tris base (10.0 mM) was added into each well to dissolved the SRB dye and measured the optical density (O.D.) value at 570 nm using microplate reader (Tecan Sunrise^TM^, Switzerland). The survival or viability of cell was calculated as follows:

$$\text{Cell survival }(\%)=\frac{\text{Treated cells OD value}}{\text{Untreated cells OD value}}\;\times 100$$

The IC_50_ values were calculated after 24, 48 h treatment using calculator (Quest Graph™).

#### 2.4.5. Evaluation of *in vitro* cell apoptosis

Apoptosis induced by nanocubosomes in HCT-116 cells were detected by flow cytometery (Shao et al., 2019). HCT-116 cells (1 × 10^5^/well) were cultured in 12-well plate and allow to grow (85 ± 5% confluency) in 5% CO_2_ incubator for 12 h. Afterwards, the cells were incubated with CTNCs and mixture of free Cur and TMZ at concentration of 5.0 µg/mL for 12 h, followed by staining with cell apoptosis analysis kit (Bio-Rad, Wuhan, China) according to manufacturer’s instructions and detected the apoptosis by flow cytometry (BD LSRFortessa, San Jose, CA 95131 USA).

#### 2.4.6. Assessment Pgp expression

The expression of P-gp in HCT-116 colon cells were evaluated by western blotting after incubation with vehicle, TMZ, Cur and CTNCs. Briefly, the HCT-116 cells (1 × 10^5^/well) were seeded in a 6-well plate and incubated for 12 h. Next, the cells were treated with TMZ (3.7 µg/mL), Cur (5.0 µg/mL) and CTNCs containing TMZ (3.7 µg/mL) and Cur (5.0 µg/mL) for 24 h. Afterward, the treated HCT-116 cells were lysed and extract the proteins by using RIPA buffer (Bio-Rad, Wuhan, China) and protease/phosphatase inhibitor cocktail at (1:100 working dilution). The cell lysate was collected and centrifuge at 10,000 ×g, 4 °C for 10 min. The protein concentration in supernatant was determined by Standard BCA method. Afterwards, western blot analysis was performed according to a standard method (Shao et al., 2019).

### 2.5. Statistical analysis

To validate the accuracy and reproducibility of the data, all the experiments were performed in triplicate independently. The data are presented as the mean ± SD. The statistical difference was calculated by Student’s *t-tests* (OriginPro8 software, OriginLab Corporation, Northampton, MA, USA). The *p values < 0.05 and ^#^*p* values > 0.05 were considered statistically significant and insignificant, respectively.

## 3. Results and discussion

### 3.1. Preparation and characterization of nanocubosomes

**Figure 1.**
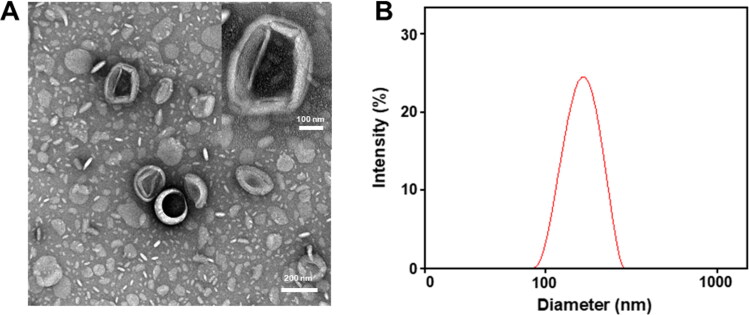
Characterization of CTNCs. (A) Surface morphology by TEM. (B) Hydrodynamic particle size by DLS.

Using modified emulsification method, nanocubosomes co-loaded with dual anticancer drugs were successfully fabricated for synergistic colon cancer therapy. The prepared CTNCs exhibited a nearly cubic polyangular geometry with core diameter of 120.5 ± 10.2 nm as examined by TEM, as described in Figure 1A. Further, the CTNCs displayed an average hydrodynamic particle size of 150.8 ± 5.6 nm with a narrow size distribution (PDI = 0.152), and surface zeta potential of analyzed by DLS as described in Figure 1B. The hydrodynamic particle size of CTNCs is slightly bigger as compared to blank nanocubosomes (Table 1), this increase in size might be induced by the drug loading into nanocubosomes.

**Table 1.** Average diameter, PDI and Zeta potential value of BNCs and CTNCs.

Samples	Avg. diameter (nm)	Polydispersity (PDI)	Zeta potential (mV)
BNCs	130.2 ± 4.2	0.162	−23.5 ± 0.5
CTNCs	150.8 ± 5.6	0.152	−35.2 ± 2.3

Nanoformulations with a small particle size (< 200 nm) exhibited enhanced cellular uptake and cell penetration compared to bigger size (Goodman et al., 2007). The hydrated PS of CTNCs was slightly bigger than its core diameter, as CTNCs were surrounded hydration layer in aqueous dispersion. Overall, the average PS of CTNCs was less than 200 nm, which appeared promising for enhanced cellular uptake. Furthermore, the CTNCs exhibited a zeta potential of −35 ± 2.3 mV, indicating that they bear sufficient charges on their surface which actively inhibited their aggregation in physiological environment and subsequently enhanced the stability of CTNCs (Table 1).

**Figure 2.**
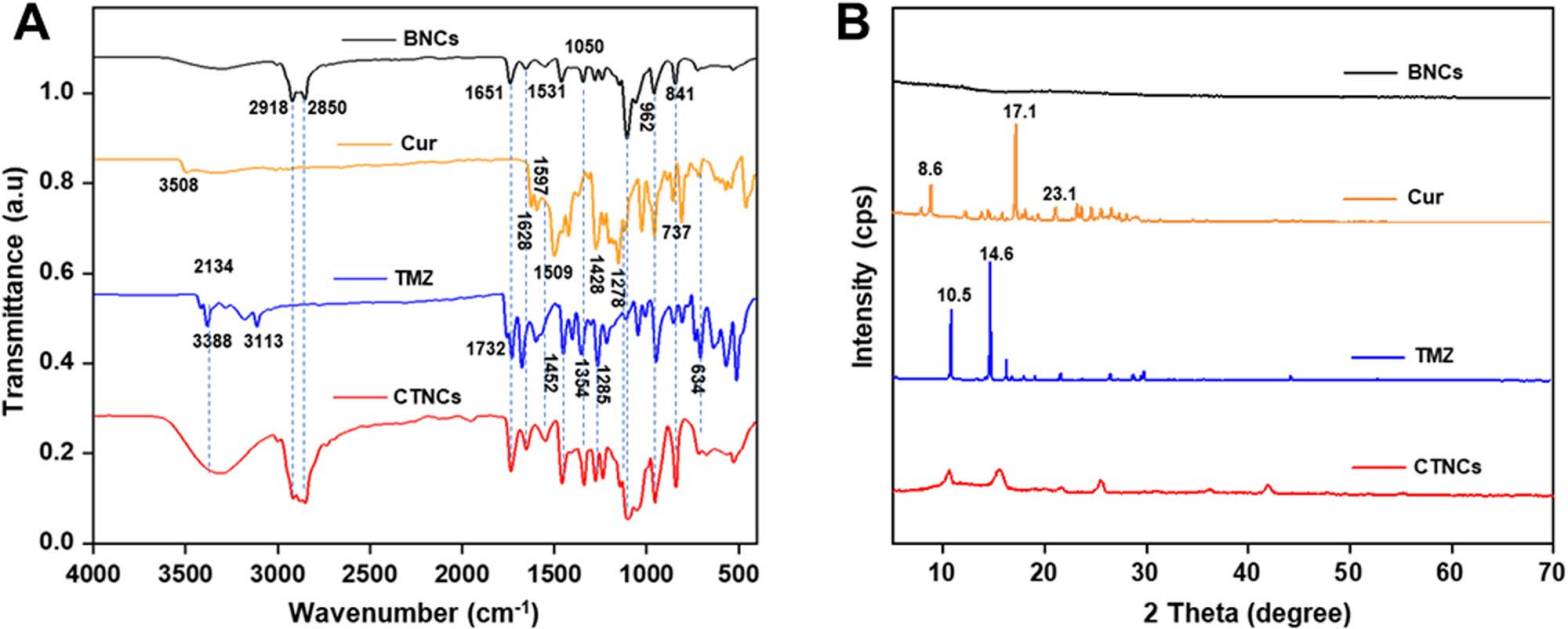
Physico-chemical Characterization of Cur, TMZ, BNCs and CTNCs. (A) FTIR spectrum. (B) XRD pattern.

The successful loading of Cur and TMZ into CTNCs were measured by UV spectroscopic and FTIR analysis. CTNCs exhibited DL of (6.82 ± 2.03% and 9.65 ± 1.53%) and EE of (67.43 ± 2.16% and 75.55 ± 3.25%) for Cur and TMZ, respectively. The FTIR spectrum of blank nanocubosomes, Cur, TMZ and CTNCs were depicted in Figure 2A. The FTIR spectrum of pure Cur show the representative peaks at 1024 cm^−1^ (C–O–C vibrations), 1278 cm^−1^ (C–O vibrations), 1428 cm^−1^ (C-H bending vibrations), 1509 cm^−1^ (C = C and C = O vibrations), 1597 cm^−1^ (stretching vibrations of benzene ring), 1628 cm^−1^ (C = C stretching, aromatic moiety) and 3508 cm^−1^ (–OH vibrations) (X. Chen et al., 2015). The IR spectrum of TMZ show attributed peaks at 3424 cm^−1^ and 3388 cm^−1^ (N-H stretching vibration), 3113 cm^−1^ and 3183 cm^−1^ (C-H stretching vibration); 1755 cm^−1^ and 1730 cm^−1^, (C = O stretching vibration), 1670.21 cm^−1^ (C = C stretching vibration}; and 1361 cm^−1^ and 1179 cm^−1^ (C-N stretching vibration) (Łaszcz et al., 2013). FTIR spectrum BNCs also show various characteristic peaks of GMO at 2861 cm^−1^ and 2882 cm^−1^, F127 peaks at C-H stretching peaks 842 cm^−1^, 961 cm^−1^, 1147 cm^−1^ and albumin peaks at 1531 cm^−1^ and 1651 cm^−1^ (Han & Wang, 2016; Motevalli et al., 2019; Kavya & Jobin, 2020). The FTIR spectrum of freeze-dried CTNCs showed all of the representative peaks of Cur, TMZ, GMO, F127 and albumin, retaining Cur, TMZ and other constituents of CTNCs with no apparent structural changes during nanocubosomes formulation. Furthermore, the broadening, shifting, or vanishing of some representative peaks demonstrate the CTNCs formation.

X-ray diffractograms (XRD) of BNCs, Cur, TMZ and CTNCs were shown in Figure 2B. XRD pattern of Cur and TMZ showed its attributed sharp and intensive peaks at 2θ values of 8.6, 17.1, 23.1 for Cur and 10.5°and 14.6° for TMZ, respectively indicated the crystalline nature of drugs (Kannamangalam Vijayan et al., 2021; Kudarha & Sawant, 2021). The XRD spectrum of BNCs did not showed any peak indicating its amorphous behavior. However, the diffractograms of CTNCs show attributed peaks of Cur and TMZ with much lower intensity. This decrease in intensity might be caused by the conversion of crystalline form of Cur and TMZ into amorphous after encapsulating in nanocubosomes. The conversion of crystalline form of Cur and TMZ into amorphous after encapsulating in nanocubosomes may also enhanced the solubility and bioavailability of Cur and TMZ as amorphous drugs are more soluble compared to crystalline drugs.

**Figure 3.**
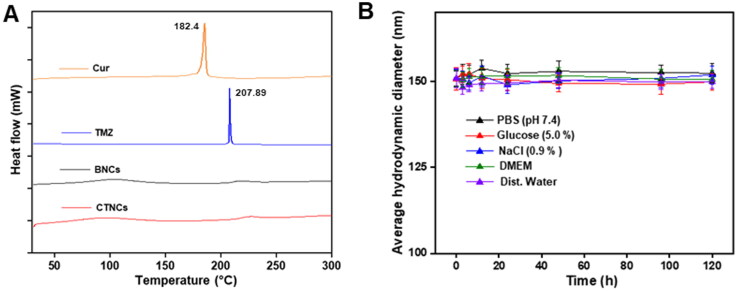
**(A)** DSC analysis of Cur, TMZ, BNCs and CTNCs. **(B)** Colloidal stability of CTNCs in different physiological medium for 120 h. Data are presented as mean ± SD.

DSC thermograms of Cur, TMZ, BNCs, and CTNCs are depicted in Figure 3A. Cur and TMZ showed its characteristic melting point peaks at 182.4 °C and 207.89 °C, respectively indicating its crystalline nature (Jain et al., 2016; Kannamangalam Vijayan et al., 2021). However, the thermogram of CNTCs did not display the sharp melting point peaks of Cur and TMZ, indicated that the drugs changed into amorphous state after encapsulation in CTNCs.

The colloidal or dispersion stability is a vital parameter, as nanoscale sized particles dispersed in physiological medium have high proclivity to agglomerate, and the formed of bigger aggregates. So, the colloidal stability of CTNCs were evaluated upto 120 h (5 days) stored at 4 ◦C in different physiological medium, i.e. PBS (pH 7.4), Dist. water, DMEM, NaCl (0.9%) and glucose (5.0%) by measuring the hydrodynamic diameter at specified time periods. The obtained results revealed that no visible change was observed in average hydrodynamic diameter of CTNCs after incubating with different physiological over a period of 120 h at 4 °C, depicted in Figure 3B. The enhanced colloidal stability of CTNCs in dispersions might be due to the charged surface of albumin which induce high repulsive forces among nanocubosomes in dispersion (Li et al., 2021). These strong repulsive interactions prevent the nanocubosomes coming too closely in contact to each other. So, in such environment the coagulation of nanocubosomes hardly occurs.

### 3.2. *In vitro* drug release

**Figure 4.**
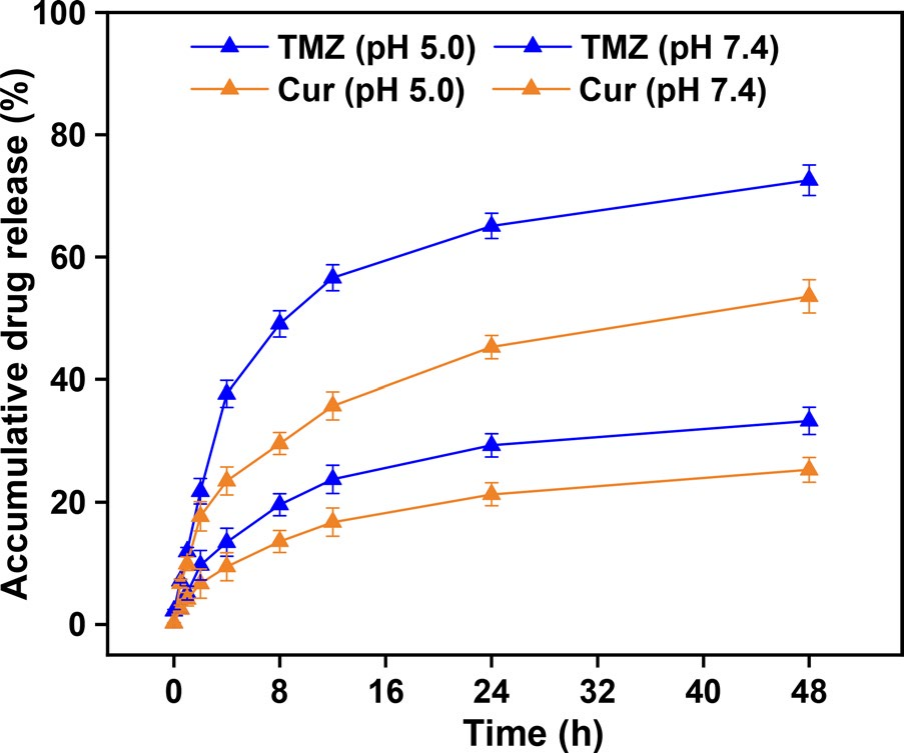
Drug release profile of Cur and TMZ from CTNCs at pH 5.0 and pH 7.4. Data are expressed as mean ± SD.

Next, we tested the release of Cur and TMZ from the CTNCs at pH 5.0 and pH 7.4, mimic the environment encountered by the CTNCs upon reaching to the lysosomal compartment. The acidic pH of lysosomal compartment induced the conformational changes in the structure of GMO and albumin instigated the release of TMZ and Cur from the CTNCs with first order release kinetics. As depicted in Figure 4, Cur and TMZ release at pH 5.0 was about 2-fold higher compared to that release at pH 7.4 and showed a higher drug release at pH 5.0. The accumulative Cur and TMZ release at pH 5.0 (53.5 ± 2.7% and 72.5 ± 2.5%) and pH 7.4 (25.2 ± 2% and 33.2 ± 2.2%) over 48 h, respectively. Moreover, a slight burst release was observed for both drugs at pH 5.0 within the first 2–4 h, which might be attributed to surface adsorption of drugs on CTNCs. The release of rate both drugs increased at pH 5.0, however, Cur exhibited a slower release kinetics to TMZ. The slower or lesser release of Cur might be due its strong hydrophobic nature and strong interaction with the lipophilic regions of GMO and albumin compared to TMZ (Chen (Dreis et al., 2007; Q. Chen et al., 2015).

### 3.3. Cellular uptake of nanocubosomes

**Figure 5.**
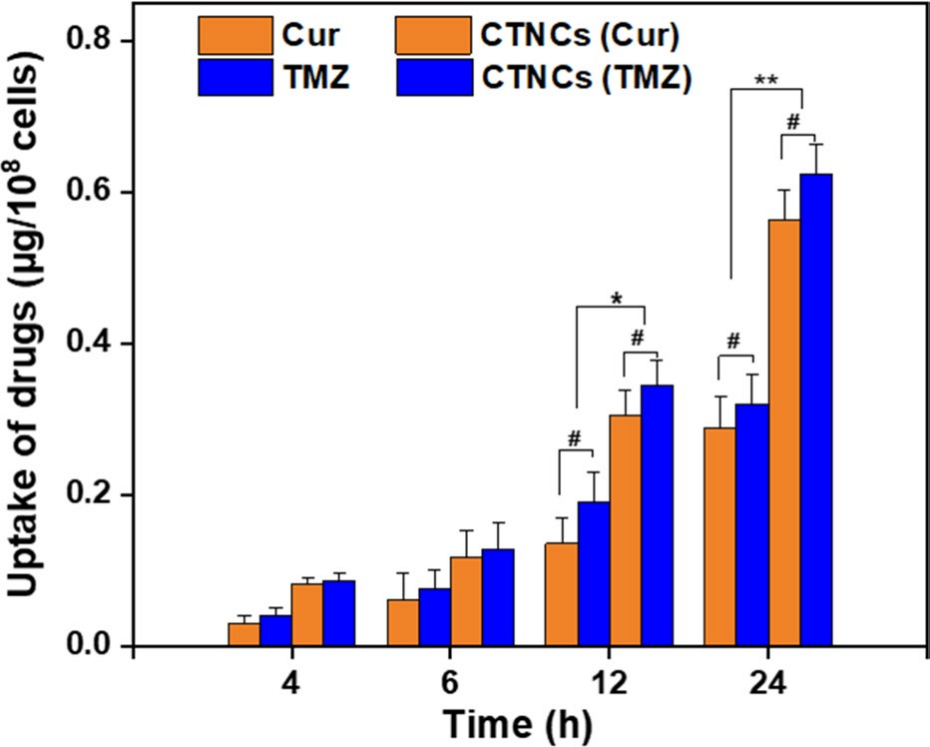
Cellular uptake of CTNCs in comparison to Cur (2.0 µg/mL) and TMZ (1.8 µg/mL) for 4, 6, 12 and 24 h. Data are expressed as a mean ± SD, **^#^** *P* value > 0.05 and *P value < 0.05, **P value < 0.01 were considered statistically insignificant and significant, respectively.

To evaluate the capacity of CTNCs to transport Cur and TMZ into the interior of cells, the cell uptake of Cur and TMZ was determined in HCT-116 colon cancer cells. As depicted in Figure 5, the cell uptake of Cur and TMZ by HCT-116 cells significantly increased (*P* < 0.05), as the incubation time of CTNCs increased from 4 to 24 h, compared to free Cur and TMZ. So, the cell uptake reached to its maximum level after 24 h of treatment with CTNCs at concentration of 2.0 µg/mL and 1.8 µg/mL with respect to Cur and TMZ, respectively.

CTNCs demonstrated time- dependent cellular uptake and exhibited 3-fold and 2.5-fold increase in cellular uptake as compared to free Cur and TMZ, respectively, after treatment with 2.0 µg/mL and 1.8 µg/mL for 24 h. The increase in cell uptake might be associated with the small particle size and albumin stabilization which improved colloidal stability and dispersion in several dissolution media (Gu et al., 2015). Indeed, the small particle size and colloidal stability provide more chances for CTNCs to interact with HCT-116 cancer cells to be internalized in comparison to free Cur and TMZ.

### 3.4. Endocytosis and intracellular distribution of CTNCs

**Figure 6.**
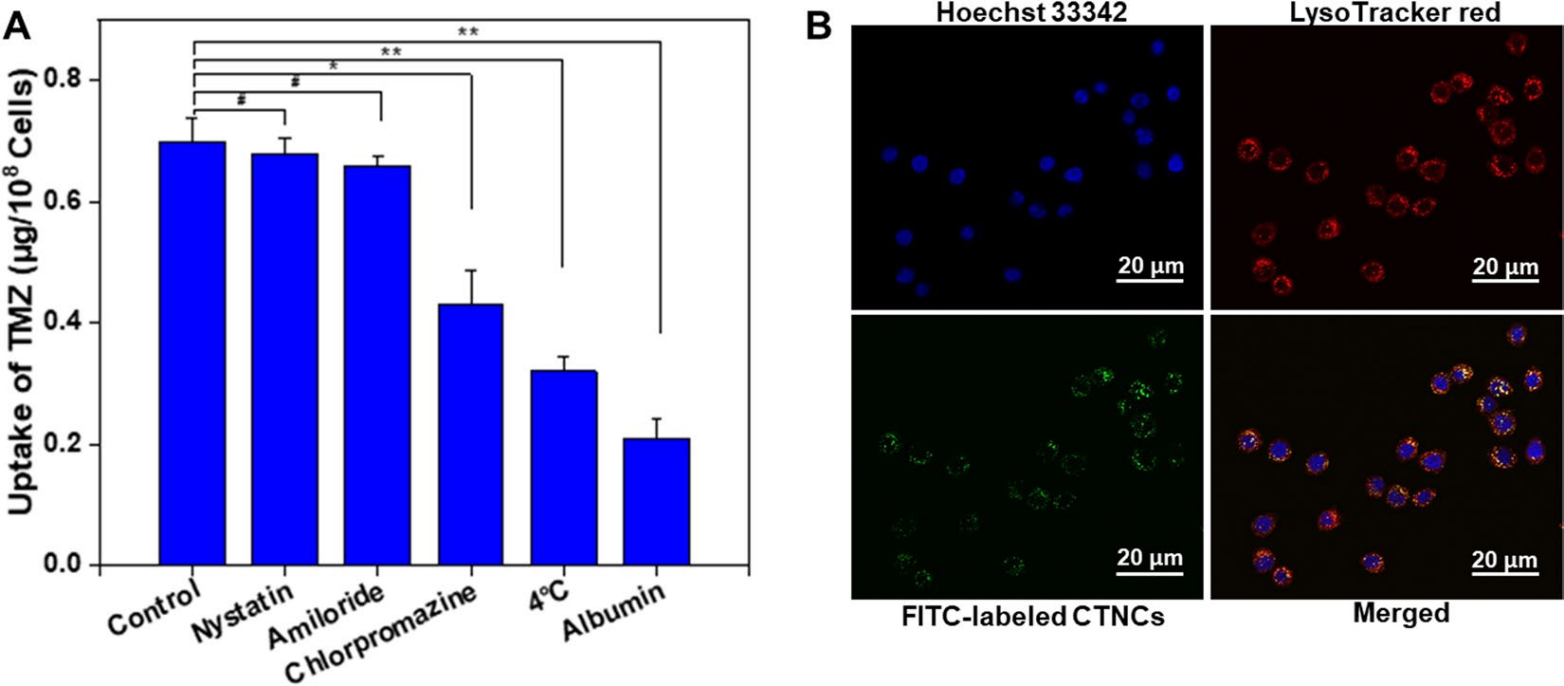
(A) Mechanism CTNCs internalization to HCT-116 cells pretreated with various inhibitors. (B) Intracellular distribution of CTNCs in HCT-116 analyzed by confocal microscope. Data are expressed as a mean ± SD, **^#^** *P* value > 0.05 and *P value < 0.05, **P value < 0.01 were considered statistically insignificant and significant, respectively.

The endocytic pathway or internalization mechanism of CTNCs were evaluated in HCT-116 cells. After treatment with various inhibitors, it was observed that chlorpromazine caused 58.68% decrease in the uptake of CTNCs (TMZ), compared to untreated cells (control group: without inhibitors), as shown in Figure 6A. However, amiloride and nystatin have no visible effect on the internalization of CTNCs, demonstrating clathrin-mediated endocytosis (Figure 6A). Moreover, incubation at 4 °C instigated 64.29% decrease in the internalization of CTNCs, as compared to cells incubated at 37 °C, indicating that internalization mechanism involves temperature or energy dependent active transport. Interestingly, the pretreatment of albumin causes 70.0% decrease in uptake of CTNCs (Figure 6A), demonstrating an albumin receptor-triggered uptake of CTNCs, since the pretreatment of albumin repressed the receptors on cancer cells (Haroon Iqbal et al., 2021; Iqbal et al., 2022). Moreover, we also evaluated the intracellular distribution of FITC-labeled CTNCs in LysoTracker Red stained HCT-116 cells. CTNCs with green fluorescence showed 87% co-localization with the lysosomes after incubation for 6 h (Figure 6B), suggesting that CTNCs uptake into lysosomes occur via clathrin-mediated endocytosis. Our results agree with previously reported literature, which showed that albumin nanoparticles enter into HCT-116 cells via albumin receptors and clathrin mediated endocytosis (Park et al., 2019).

### 3.5. Cytotoxicity of CTNCs

**Figure 7.**
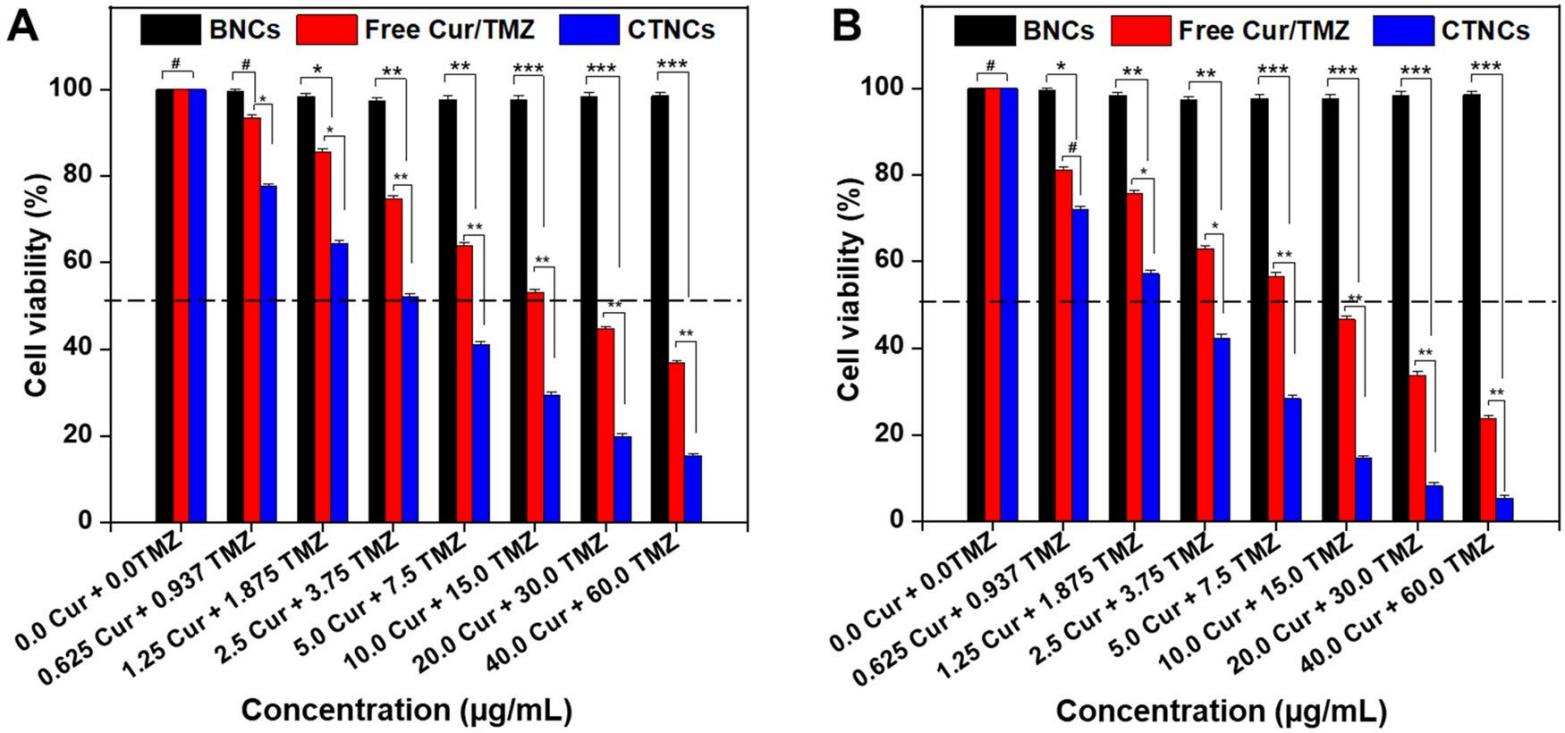
Viability of HCT-116 cells after treatment with BNCs, mixture of free Cur/TMZ and CTNCs. (A) 24 treatment. (B) 48 h treatment. Data are expressed as mean ± SD. **^#^** *P* value > 0.05 and *P value < 0.05, **P value < 0.01, ***P < 0.001 were considered statistically insignificant and significant, respectively.

The *in vitro* cytotoxicity of CTNCs against HCT-116 cells were evaluated by SRB assay. After treatment with mixture of free Cur and TMZ and CTNCs for 24 h and 48 h, HCT-116 cancer cell growth was inhibited. As shown in Figure 7A, CTNCs significantly inhibited (up to three-fold) the growth of HCT-116 cancer cell in concentration dependent manner after 24 h of treatment as compared to a mixture of free Cur and TMZ, with IC50 with 6.16 µg/mL and 11.69 µg/mL, respectively. In addition, CTNCs further decreased the cells viability with IC50 value (4.48 µg/mL) compared to with IC50 value of mixture of free Cur and TMZ (8.25 µg/mL) when treated for 48 h Figure 7B, suggesting that CTNCs exhibited both time and concentration dependent cytotoxicity against HCT-116 cells. Hence, the improved cytotoxic activity of CTNCs might be attributed small particle size and albumin which improved the receptor-mediated internalization CTNCs, considering that HCT-116 cells uses albumin as a source of nutrient. The enhanced cytotoxic efficacy of CTNCs, compared to a mixture of free Cur and TMZ confirmed the higher cellular uptake and receptor-mediated endocytosis of CTNCs, which is highly advantageous for synergistic anticancer activity. In addition, the higher cytotoxic effect of CTNCs may be explained by hypersensitization of tumor cells due to the presence of the Pluronic® copolymer (Mehanny et al., 2016b). Moreover, the HCT-116 cells incubate with BNCs displayed high cell viability, demonstrating that BNCs have no cytotoxic effect against the HCT-116 cells.

### 3.6. *In vitro* cell apoptosis

**Figure 8.**
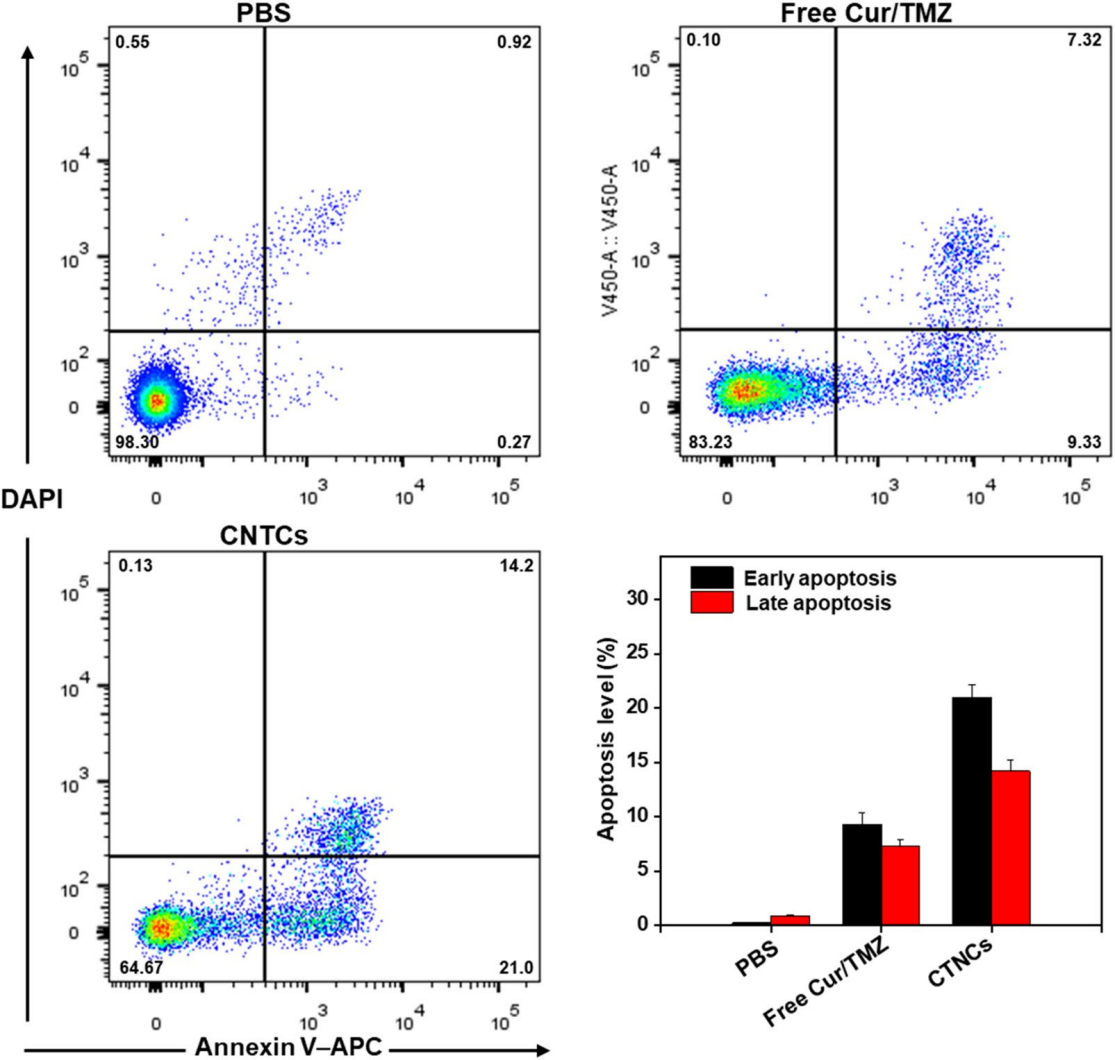
Apoptosis level in HCT-116 cells after treat with free Cur/TMZ and CTNCs (Cur 4.0 µg/mL and TZM 6.0 µg/mL) for 24 h. The apoptosis level (%) were measured by cell apoptosis assay kit and flow cytometery.

The *in vitro* cell apoptosis was investigated by annexin V-APC/DAPI assay. Live cells, early stage apoptotic, late stage apoptotic and necrotic cells were quantitatively analyzed by annexin V-APC/DAPI double staining assay; Annexin V–APC can easily penetrate through viable cells, while DAPI only stained dead cells. As shown in Figure 8, the control group has high percentage (approximately 98.3 ± 1.2%) of viable cells, localized in the lower left quadrant. However, the percentage of live cells decreased to 83.23% and 64.67% after treatment with mixture of free Cur/TMZ and CTNCs respectively, and subsequently the percentage of apoptotic cells increased to 35. 3% in CTNCs treated cells, when compared with apoptotic cells (16.65%) treated mixture of free Cur/TMZ, indicating the synergistic effect CTNCs against HCT-116 colon cancer cells.

### 3.7. Pgp expression assessment

**Figure 9.**
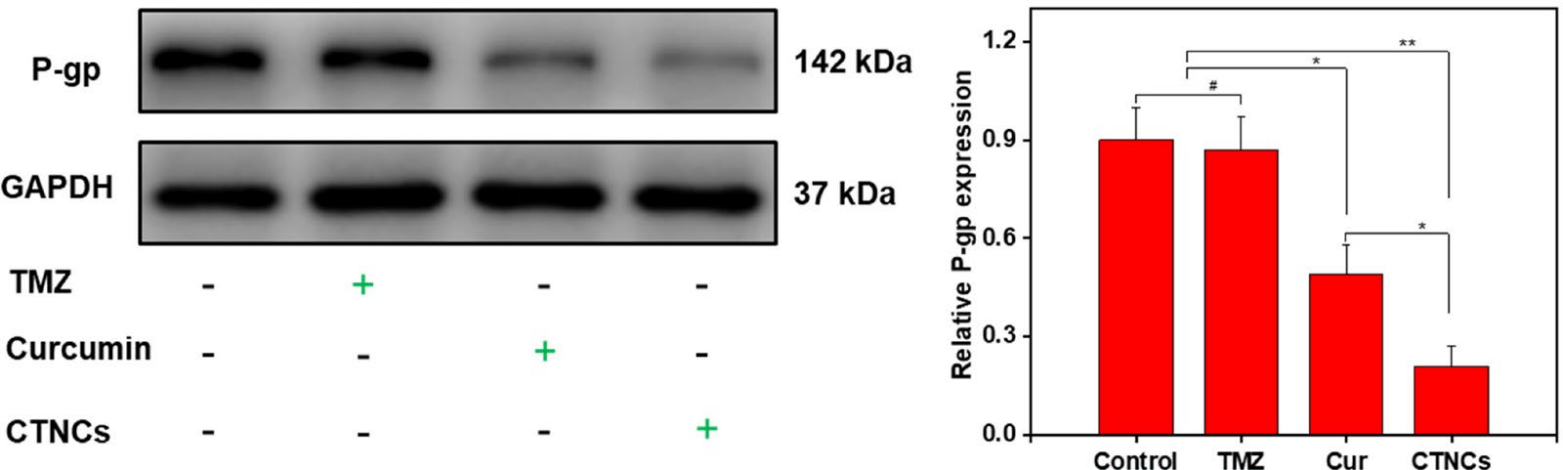
Expression of P-glycoprotein (142 kDa) in HCT-116 cells treated with PBS (control), TMZ (3.7 µg/mL), Cur (5.0 µg/mL) and CTNCs (TMZ 3.7 µg/mL and Cur 5.0 µg/mL) for 24 h.

Finally, we evaluated the P-gp expression in HCT-116 cells treated with CTNCs, free Cur and TMZ for 24 h. Untreated cells were used as control group. As shown in Figure 9, P-gp expression significantly downregulated in cells treated with CTNCs and Cur compared to TMZ treated cells., indicating that Cur is responsible for downregulation P-gp expression. However, CTNCs treated cells show lower P-gp expression as compared to free Cur, which intimated that Cur can downregulate the P-gp expression strongly when deliver to lysosomes. Our results are consistent with recently reported albumin nanoparticles co-loaded with Cur and doxorubicin for blocking ATT in breast cancer cells (Motevalli et al., 2019). In addition, the augmentation of the anticancer activity of CTNCs via Pluronics®, could be attributed to several factors including enhanced cellular uptake into malignant cells by membrane fluidization, inhibition of several drug resistance mechanisms (P-gp efflux, drug sequestration within cytoplasmic vesicles) and ATP depletionin MDR cancer cells.

## 4. Conclusion

In current study, we successfully prepared nanocubosomes that simultaneously deliver Cur and TMZ to the tumor cell to exert synergistic effect for overcoming ATT in colon cancer. The self-assembled nanocubosomes exhibited small particle size, enhanced drug loading, excellent colloidal stability, sustained drug release profile, enhanced cellular uptake and synergistic *in vitro* cytotoxicity. Importantly, the anticancer efficacy of TMZ against HCT-116 colon cells could be upsurge by Cur due to blocking the transport of TMZ through P-gp pumps from the cells by inhibiting P-gp expression, which eventually induced strong anticancer effect. In short, this study exploited the importance of simultaneous co-administration of chemosensitizers and chemotherapeutic drugs to enhance the therapeutic efficacy of anticancer drugs.
